# Pulmonary arterial pressure: a look into the future

**DOI:** 10.1186/1532-429X-17-S1-T2

**Published:** 2015-02-03

**Authors:** Ronald B Williams, Mark Doyle, Geetha Rayarao, Robert W Biederman

**Affiliations:** 1 Cardiology, Allegheny General Hospital, Pittsburgh, PA, USA

## Background

Pulmonary Hypertensive (PH) is an incurable , and often fatal disease. Currently a patient's treatment requires periodic office visits to evaluate their progression of right heart failure and increased pulmonary arterial pressures associated with this disease. Invasive and serial right heart catherization (RHC) is the current accepted method for assessing pulmonary arterial pressures and objectively tracking a patient over time. There is an obligate morbidity and mortality associated with serial RHC's. Further, with an estimated >1000 new diagnoses yearly and their requirement for serial RHC's, a novel approach to measuring pulmonary arterial pressures that might have added utility beyond a simple manometer is warranted.

## Methods

Use of a unique implantable device, CardioMEMs®, recently approved by the FDA (Feb 2014), permits real time evaluation of a patient's pulmonary pressures on a minute-by-minute basis. Novel integration of CMR data with the manometer capacity permits comparison and tracking of pulmonary pressure changes under certain applied stressors.

**Figure 1:**
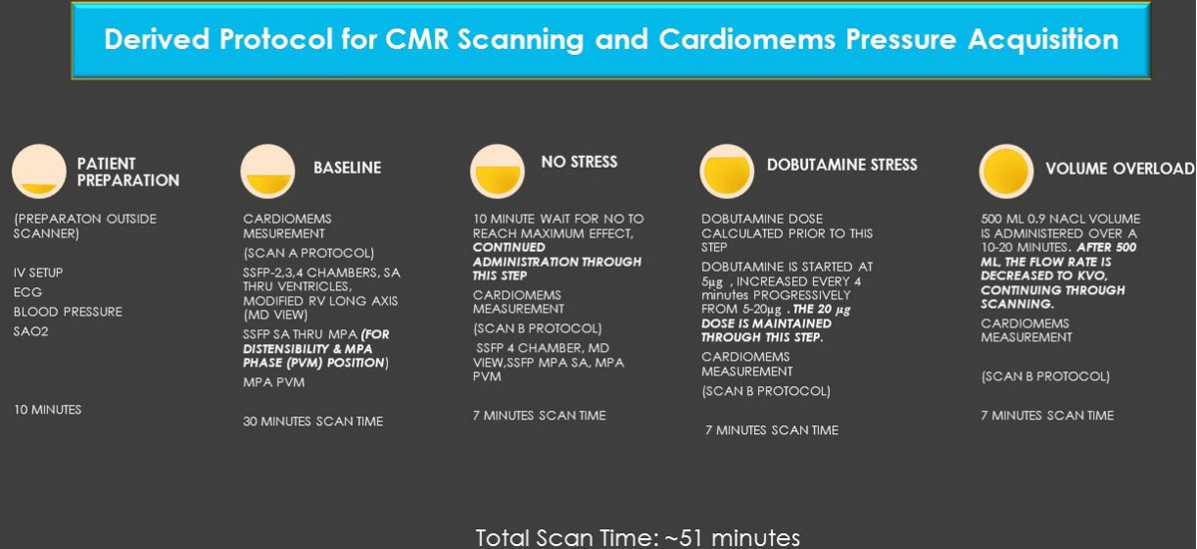


Patients with the CardioMEMs implant, inserted via a RHC in the left pulmonary artery branch, are evaluated following a complex, derived protocol schema (Figure 1).

**Figure 2:**
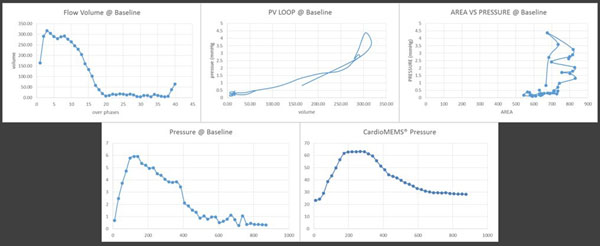


Direct comparison of data acquired from the baseline, nitric oxide (iNO), dobutamine and volume challenge were compared to determine the impact various ‘stressors' had on the right ventricle/pulmonary arteries via volume, area, flow change and the direct pulmonary pressure measured with the CardioMEMs® implant. This novel approach allows us to obtain immediate pulmonary pressure monitoring and comparative CMR data within a contracted time. A key question is: How do we obtain RV end-diastole volume rapidly? We measure the stroke volume (SV) from the MPA using phase contrast velocity imaging and the LVEF estimated from the 4 chamber (HLA)-SSFP using the Sandler-Dodge formula. Thus, Rearranging this formula, we have: (this is a key factor making the dynamic imaging faster). This now provides a new opportunity for sophisticated analytics (Figure 2).

## Results

This CMR and CardioMEMs® showed strong pressure correlation ( 0.91).

## Conclusions

A comprehensive evaluation of the RV/PA system is possible using CMR and the implanted CardioMEMs® pressure measuring device. Given the many stressors, it now possible to use CMR in a most efficient manner to obtain heretofore invasively-mandated clinical information in completely non-invasive manner.

## Funding

Internal funding.

